# A qualitative exploration of English black adults’ views of strength and balance activities in mid-life

**DOI:** 10.1186/s12889-022-14382-4

**Published:** 2022-11-17

**Authors:** Nick Cavill, Gill Cowburn, Russell Jago, Charlie Foster

**Affiliations:** 1 Centre for Exercise, Nutrition and Health Sciences, School for Policy Studies, University of Bristol, 8 Priory Road, BS8 1TZ Bristol, England; 2 NIHR BRC Nutrition Theme, UHBW Education and Research Centre, Level 3, Upper Maudlin Street, BS2 8AE Bristol, England; 3 Population Health Sciences Bristol Medical School, University of Bristol, Bristol, England

**Keywords:** Physical activity, Strength, Balance, Retirement, Black, Ethnic minority, Qualitative

## Abstract

**Background:**

Public health guidelines state that all adults should undertake muscle and bone strengthening and balance training activities at least twice a week to support their physical function and maintain independent health. This is intended to maintain strength in adulthood and offset natural declines in bone density and muscle mass. Most older adults do not meet this guideline with low levels of compliance among older black people. This study explored the experiences of physical activity relating to strength and balance activities, amongst black men and women living in England, aged 50–70.

**Methods:**

Participants were recruited by phone via a network of research recruitment specialists across England. In-depth qualitative interviews were conducted with 25 black people aged 50–70 living in England. An inductive thematic analysis was conducted.

**Results:**

We found there was only a very general understanding of the importance of maintaining body strength and balance, and low salience: strength and balance activities were not seen to be an important part of participants’ lives. Most participants only wanted to be strong enough to get on with ‘normal life’ and not to build strength or balance. Participants aged 50–70 were likely to think they were too young to worry about strength and balance, which tended to be mentioned only if someone had experienced a problem. Participants reported that NHS staff, especially physiotherapists are a key source of information on the topic and could therefore be useful in future prevention strategies.

**Conclusion:**

Public health recommendations stress the importance of increasing participation in regular strength and balance activities as people age, to reduce the risk of falls and maintain independence. This study has shown that among the black middle-aged adults we interviewed, the knowledge and salience of this message is low. Public health approaches should be taken to communicate the importance of enhancing strength and balance as people approach older age, including communication and education programmes led by health professionals, who were viewed with authority amongst these participants.

## Background

Since the mid 1990s epidemiological evidence has shown that regular participation in different types of physical activities is consistently associated with reducing the risk of all-cause mortality, stroke, cardiovascular disease, frailty, falls and range of cancers. Participation also provides a range of mental health benefits at all ages across the life course [1].

Global and national guidelines for physical activity recommend that older adults (> 65 years) should undertake 150–300 min of moderate-intensity, or 75–150 min of vigorous-intensity physical activity, or some equivalent combination of moderate-intensity and vigorous- intensity aerobic physical activity, per week [1, 2]. In addition, public health guidelines state that all adults should undertake muscle and bone strengthening and balance training activities at least twice a week to support their physical function and maintain independent health. This is intended to maintain strength in adulthood and offset natural declines in bone density and muscle mass. Balance training is specified as a recommendation for older adults over 65 years and older in the UK CMO Guidelines [1].

Recent evidence has clearly shown the importance of strength and balance activities to improve bone and muscle health and physical capacity at any age, and - in middle and older age - to maintain and improve function and reduce all-cause and cardiovascular mortality [3].

There are particular ages where muscle strengthening and balance activities are most important. From 18 to 24 years these activities maximise bone and muscle gains, while between 40 and 50 years they maintain strength and slow the natural decline. In over-65 years they preserve strength and maintain independence. Strength and balance exercise may also improve future health outcomes at specific life events [4]. These are events that may prompt adults to increase their sedentary behaviour or reduce their physical activity, leading to loss of muscle function. Such events include pregnancy, menopause, onset of/on diagnosis of disease, retirement, on becoming a carer or following hospitalisation [5].

Everyday activities that challenge balance and strength include standing up from sitting in a chair, walking up and down stairs, climbing up a ladder, carrying shopping or picking up a toddler. Types of physical activities or sports that encourage these improvements include racquet sports, yoga, Tai Chi, as well as resistance training and aerobics and circuit training [3].

Recent UK surveillance studies have reported that most adults and especially older adults do not meet current UK physical activity guidelines, particularly when reporting proportions who achieve both the aerobic and strength and balance requirements [6] [7]. There are inequalities in participation in physical activity: participation declines with age [8]; is lower among women compared with men [6] ; and lower among many ethnic minority communities compared with the white population [9] [10]. These inequalities in activity also reflect differences in morbidity across these groups and alongside recent studies of participation across and post COVID19 lockdown, places the UK a worsening situation and widening inequalities across all of these groups. People from ethnic minority communities tend to be under-represented in research studies [11], including on physical activity [12]. Our recent systematic review identified a lack of any UK based studies looking at understanding black minority groups experiences in relation to strength and balance activities [13]. We therefore set out to explore and understand the experiences of physical activity relating to strength and balance activities, amongst black men and women living in England, aged 50–70. We defined balance activities as everyday activities that involve individuals balancing themselves while moving, lifting or carry things and strength activities as those where individuals lift or carry items or perform actions that use their muscles. We chose the age range 50–70 to explore the extent to which people think about strength and balance activities as they approach – and possibly prepare for – older age. This complements recent studies on physical activity and active travel among this age group [14].

## Method

Ethical approval for the research was provided by the University of Bristol School for Policy Studies Research Ethics Committee (Underpin 2 (SPSREC/20–21/139). Qa Research, an independent social research company, recruited and conducted semi-structured telephone interviews with 25 participants in January 2022. Participants were recruited using the Qa Research network of research recruitment specialists across England, who hold a database of local people who have consented to be approached to take part in social and market research. Potential participants were contacted via telephone and provided informed written consent prior to inclusion. Interviews were conducted at a convenient time for participants and took no longer than 30 min. An interview guide (appendix 1) was used to lead the discussion, but participants were encouraged to talk openly about each topic. Each participant received a payment of £40 on completion of the interview, as an acknowledgement of their contribution to the study. Interviews were audio recorded (with permission) and transcribed verbatim.

We conducted a thematic analysis of the audio and transcribed interview data, using constant comparison and adopting an inductive, interpretive approach [15] [16] [17] [18] [19]. NvIvo 12 computer-assisted qualitative data analysis software was used to aid the analysis. Data were coded for descriptive and conceptual themes as they were identified and placed into an overarching framework. Concepts within and between themes were examined to explore ways to interpret and understand the data [20]. We present the findings of the study using illustrative quotes from participants, which have been edited only to remove interviewer comments and to reduce repetition.

### Participant characteristics

Table 1 provides information about the study sample. Our sample included 25 participants aged between 50 and 70 years old, selected to be from black communities living in England. Black communities included people who defined their own ethnicity as: Black/Black British; Black Caribbean; British Caribbean; or Mixed (inc Black/African/Caribbean). Most participants were still undertaking paid work in charity, education, commercial, care and local authority settings. A few participants were retired, had long-term sickness issues which prevented their employment, were actively looking for work or were studying. Several participants took on voluntary roles or acted as unpaid carers alongside their paid employment.

**Table 1:** Demographic descriptions of participants

Participant number	Sex	Age	Work status	Region	Ethnicity	Home & work circumstances	History of physical activity	Pre-existing health condition
Participant 1	Male	55	Working	Yorkshire	Black/Black British	Lives alone, cares for family member, works full time shift work	Weekly sport – football, squash, martial arts	No
Participant 2	Female	57	Retired	Yorkshire	Mixed (inc Black/African/ Caribbean)	Has partner & grown up children, retired following redundancy	Regular activity – aerobics, Zumba, Badminton	No
Participant 3	Female	57	Retired	Yorkshire	Black/Black British	Lives alone, voluntary work	Enjoyed sport at school, regular walking	No
Participant 4	Female	56	Working	London	Black Caribbean	Lives with partner & grown up children, works in charity sector	Enjoyed sport at school, gym membership, dancing & regular walking	No
Participant 5	Male	55	Looking for work	South East	Black British	Lives with partner & young child, voluntary football coach	Enjoyed sport at school, gym membership, football and regular running & walking,	No
Participant 6	Female	63	Working	South East	Mixed (inc Black Caribbean)	Lives alone, grown up children, works in commercial sector	Previous gym membership & personal trainer, enjoyed Zumba & walking	Yes
Participant 7	Female	60	Looking for work	London	Black Caribbean	Lives with child	Enjoyed tennis, badminton, dancing, has gym membership, regular walking	No
Participant 8	Female	57	Working	London	Black African	Lives with partner, elderly mother, has grown up children. Works in the social housing sector	Previous gym attendance, regular walker	Yes
Participant 9	Female	55	Working part-time	South West	Black British	Lives alone, works in the commercial sector	Active childhood, previous regular gym attendance, cycling & regular walking	Yes
Participant 10	Female	55	Working part-time	South West	Dual Heritage (Italian/Caribbean)	Lives with grown up children, works in the education sector	Previously regular cycling & walking	Yes
Participant 11	Male	55	Working	South West	Dual Heritage (British/Caribbean)	Lives with partner, works in the commercial sector	Active childhood, regular swimming, cycling, gym	No
Participant 12	Female	58	Long term sick	East Midlands	British Caribbean	Lives alone, grown up children, previously worked in the care sector	Previous regular attendance at gym, regular walker	No
Participant 13	Male	63	Working part-time	East Midlands	British Caribbean	Works in local government sector	Previously active – athletics, regular running, swimming and gym sessions	Yes
Participant 14	Male	64	Working	London	Black African	Lives alone, works in the commercial sector	Active childhood, regular running and walking	No
Participant 15	Female	58	Working	West Midlands	Black Caribbean	Lives with teenage child, works in the care sector	Active childhood, regular gym and walking	No
Participant 16	Male	55	Long term sick	West Midlands	Black Caribbean	Has children, previously worked in the commercial sector	Active childhood, regular cycling, gym attendance	Yes
Participant 17	Female	55	Working part-time	West Midlands	Black Caribbean	Has grown up children, works in the care sector alongside caring role	Active childhood, regular yoga and walking	Yes
Participant 18	Female	58	Education part-time	West Midlands	Black Caribbean	Lives alone, has grown up children, voluntary work	Active childhood, limited mobility	Yes
Participant 19	Female	63	Working	East	Black		Active childhood, regular dancing, walking, yoga and Pilates	No
Participant 20	Male	59	Working	East	Black	Lives with partner, works in commercial sector	Regular walking and gym attendance	No
Participant 21	Female	64	Working	West Midlands	Black Caribbean	Lives with grown up child, works in education sector	Active childhood, regular walking	No
Participant 22	Female	56	Working	North West	Black Caribbean	Lives with partner and teenage children, carer for family members	Active childhood, regular swimming, gym, dancing and walking	Yes
Participant 23	Female	57	Working	North West	Mixed Black African/Caribbean	Lives with grown up child and teenage foster child, works in care sector	Active childhood, regular walking	Yes
Participant 24	Male	60	Working	North West	Black British/Caribbean	Has a grown up child, works in education sector	Active childhood, regular gym and badminton	No
Participant 25	Female	65	Retired	South East	Black British	Lives alone, voluntary work	Active childhood, regular swimming	Yes

Several participants were living with pre-existing health conditions which reduced their physical activity levels. Although some now had limited mobility, all participants had enjoyed an active lifestyle in the past – reporting that they had been ‘sporty’ at school, some to high levels, and had continued to pursue active hobbies throughout their life.

## Results

There were two broad, and distinct themes with one focusing on strength and the other balance. Within these, we explore several sub-themes that are presented below.

### Strength

#### Understanding of strength

Across this sample there was a very general and broad understanding of the concept of body strength, or the importance of developing or maintaining strength. In most discussions, strength was conflated either with exercise, or with general health and wellbeing:

*“Why is it important to maintain some strength? Well it keeps my young…Yeah. It keeps me young and it keeps me active... And it helps, it helps me keep away from the hospital and having to actually taking too much tablets or more tablets than what I need to take”* Participant 13, Male 63

*“Well it’s just about keeping movement. Otherwise, if you don’t, your body is going to seize up really…so it’s just about keeping trying to keep active*” Participant 17, Female, aged 55 years

For some respondents there was more specific understanding of the benefits of preserving muscular strength, but these were also quite general and non-specific in nature. For example:

*“Strength makes a lot of your activities easier doesn’t it? But also, you know, as you’re getting older, muscle wastage, things like that…probably less falls. Sort of many areas I think”* Participant 2, Female aged 57 years

*“Strengthen my legs because I mean you’re carrying your bodyweight on your legs so that’s an area that needs to be strengthened”* Participant 6, Female, aged 63 years

Participants mentioned strength in connection with ageing, with the notion ‘use it or lose it’ being mentioned by several respondents.

*“As we get older everything slows down…if you don’t do strength exercises you start to lose your strength as well. Yeah, muscles can get weaker…bones can become more fragile maybe or weaker”* Participant 19, Female, aged 63 years

For other people, the importance of strength was in helping specific health conditions – often recommended by a health professional (see below).

*“After the COVID I lost a lot of muscle tissue and, oh, it’s taken me weeks to kind of get my body looking like it was before”* Participant 11, Male aged 55 years

#### Judging their own strength

Most people considered strength in terms of being strong enough to do everyday tasks. This was either in generic terms – being strong enough to do the hoovering or carry shopping – or explained in terms of having a problem that demonstrated insufficient strength:

*“I went shopping at Costco and there was a pack of nine coconut waters, and I normally pick them up and stick them in the trolley without even thinking about it.. and I picked it up off the shelf and it landed on my knee. OOH!...I had to shout my son to come and help me”* Participant 23, Female, aged 57 years

#### Sources of information

Participants seemed to think that knowledge about strength was just common sense or received wisdom. If discussing a specific source of information, the most common source of knowledge about strength was through interaction with health professionals – often as a result of an injury:

*“Because of the issue that I’ve got when I go to the gym, you know, you have your selected physio person so they will give you the exercises specific for your problem. And then, of course, they will give you exercises to do at home, so it is a continuous thing really. But yes, it’s really, really been helpful”* Participant 7, Female, aged 60 years

#### What did participants say they do to enhance their strength?

As described above, people were most likely to describe strength-based aspects of daily living. These typically included.

- Carrying shopping.
- Gardening.
- Everyday housework.
- Picking up little children.

*“I bring my own shopping from the car…or if I’m really, really tired they stay in the car until the next day. Right, okay. I can manage it… if it’s too dark I take out like the things to go in the fridge and I’ll leave the rest in the car until the next day”* Participant 18, Female, aged 58 years.

*“So I think everyday living, you know, while all your bodily parts are still working, you know, functioning, I think walking up and down the steps, you know, doing everything within your home, that can be exercise in itself”* Participant 3, Female, aged 57 years.

The more active participants mentioned a wide range of strength-related activities, including:

- Rolling wheels (a device mainly for abdominal exercises).
- Exercises from YouTube.
- Weights.
- Dumbbells.
- Gym (general term).
- Yoga.
- Pilates.

One respondent mentioned the importance of protein (they had stocked up on protein supplements to help). Only one participant mentioned the contrast between enhancing strength and building visible muscle:

*“I do the weights but obviously not heavy weights because I’m not trying to get muscles, I’m just trying to keep my, you know, strength”* Participant 7, Female, aged 60 years

For many of the participants, strength activities simply did not play an important part in their life. If they could manage everyday life with the strength they had, then they felt no need to enhance strength.

*“I think I do most things so, you know, myself, so I think I just keep going. Until I feel I can’t do something, I do it”* Participant 2, Female, aged 57 years

In some cases, strength activities were not a priority compared with other types of activity:

*“I would do mainly cardio and…I was told that I’ve also got to do a bit of work on the muscles as well...but I would concentrate more on cardio because I was more interested in burning calories and losing weight* [interviewer - *So building strength wasn’t really a key kind of focus in that respect?] No. No, no. No, it wasn’t my priority, no”* Participant 24, Male, aged 60 years

#### ***Barriers to doing strength activities.***

Participants mentioned several things that stopped them from doing strength activities. The most commonly-cited barriers were:

- I don’t have the motivation.
- I can’t find the time.
- It is too far to the gym.
- I have existing injuries.
- I am in pain (including muscle pain) so can’t do more.

*“I didn’t realise until I went to the Osteopath that the effect of pulling a hoover actually is probably one of the worst things you can do for back pain”* Participant 8, Female, aged 57 years

*“I have very weak hands and wrists, and I suffer with nerve damage which gives me a lot of tingling sensations in my hands. So things that I can’t do…I don’t have much strength to pick up and twist things, so I depend a lot on my son who is here every day to help with things like that”* Participant 17, Female, aged 55 years

But for most, it appears to be the lack of salience of strength activity, and the low priority attached to it, that stops them from doing more.

*“Some people think oh I’ve tried that, done it and I don’t see any results”* Participant 3, Female, aged 57 years

## Balance

### What is balance?

The notion of balance was misunderstood by some participants, who appeared to be more familiar with it as a concept in relation to balancing their behaviour across different risk factors rather than linking it specifically to physically activity.

*“I think it’s just for your health. Obviously if you drink, say if you drink and smoke, they say that’s bad for your health, but you can balance it out by doing both. You can drink and smoke and you can still go to the gym and do plenty of exercises. My opinion is you balance it out together”* Participant 20, Male, aged 59 years

Once the specific study definition was given as a prompt (“everyday examples of activities that need good balance include: reaching up to grab something on the top shelf; walking up and down stairs; walking on uneven surfaces when outside; walking the dog; standing up from sitting in a chair; getting up from kneeling or sitting on the floor onto your feet; looking behind you while walking”), we found a range of different responses from participants. Some were surprised that issues around balance might be relevant for them. Others suggested that they had been unfamiliar with the specific concept:

*“Blimey. I think I obviously think, you know, I’m not young but I’m not old. I would seek medical or physical advice if that were the case. I mean, yeah, that would certainly come as a shock if all of a sudden my orientation went and I started to feel unsteady, yeah”* Participant 11, Male, aged 55 years

*“Bending down and picking up things, and reaching cupboards and things, you know, I do all of those, but I guess in my mind I’m not thinking of them as balancing exercises”* Participant 4, Female, aged 56 years

Some participants, having not previously considered balance activities, agreed that they might benefit from improving their balance, whereas a few participants specifically understood the link:

*“Yeah, I think maybe I need to do some… As you get older things you did without thinking, they don’t just work as they used to. So, yeah, so I think it’s a very good idea”* Participant 24, Male, aged 60 years

*“I think it is very important. I was reading something about it really helps with the muscles, especially in your legs, ones when you’re doing the balance exercise”* Participant 22, Female, aged 56 years

The participants who were familiar with the concept of balance reported receiving their understanding from various sources – common sense and general knowledge, health professionals, the internet and from awareness of local provision:

*“I think I’m sort of aware that you need to kind of keep your body going, so that’s just, I don’t know where that comes from but I just have an awareness of it”* Participant 2, Female, aged 57 years

*“I’ve actually been given, I was given some exercises by a physio. So I do some on my bed, and then some against the wall and then some against the chair. I have a routine”* Participant 8, Female, aged 57 years

*“Plenty of exercises online”* Participant 12, Female, aged 58 years

*“I’ve got a leisure centre and studios so it’s not difficult to find them”* Participant 19, Female, aged 63 years

### The benefits of good balance

Respondents were able to identify some of the benefits to improving balance. For most, these included general mental and physical health wellbeing. Some participants also linked good balance to benefits which specifically related to ageing, for example, with an ability to maintain mobility, which they directly linked to independence in older age, and a reduction in the possibility of falling.

*“Healthy body, healthy mind they say, don’t they? You know, and obviously your mobility, I’m quite lucky but as you get older your mobility is often impacted quite a lot isn’t it? So if you do a little bit while you can it helps it will probably help you later on”* Participant 2, Female, aged 57 years

*“If you’re not able to balance properly then things will become awkward and, again, you’re going to require help from others. You know, I suppose you can start to feel like you’re losing your independence because if you’re having to constantly rely on others then you’re not as independent as you would like to be”* Participant 7, Female, aged 60 years

*“I just think it’s just a way of keeping yourself as fit as you possibly can. And as they say, the older you get, you know, people tend to fall over due to their balance and things like that, hip problems”* Participant 9, Female, aged 55 years

Some participants looked to role models in their social networks to provide evidence of the benefits of maintaining balance and physical activity levels in older age:

*“It’s important that we look after ourselves… If you can walk it’s always better to walk. It’s better to keep the weight off, eat right and exercise right. It’s very, very important. Yeah. I think. Especially when you reach our age. Yeah. I mean my Mum is 96 and she’s still alive and she’s walking. She doesn’t need aids… so it can be done”* Participant 18, Female, aged 58 years

A few were less optimistic, suggesting that although they could identify potential benefits for other people, they were less likely to be able to benefit personally:

*“Because I’ve got a friend who does walking. She does her walking daily. She walks for miles and comes back and she is quite fit and active and like, yeah, I think oh maybe if I did that probably I would be the same. But I think I’ve gone too, it’s too late now to try doing things like that because I do get awful tired”* Participant 25, Female, aged 65 years

### Experiencing balance issues

Participants reported a range of experiences with their own balance. Some had no experience of any balance problems, whereas others were aware that if their balance was specifically challenged, they might have some difficulty.

*“I don’t particularly work on it but it’s not something I’ve ever had a problem with”* Participant 2, Female, aged 57 years

*“If, for instance, you were given an exercise to do based solely on your balance, maybe like, even though, you know, I try to be as active as I can, I think that would be something that you would then probably discover maybe you’re not as balanced as you thought you were”* Participant 7, Female, aged 60 years

Other participants reported specific examples of occasions when their balance had caused an issue. Several suggested that this was age-related and had resulted in them being more cautious and adapting their behaviour to reduce the risk of further problems:

*“I don’t think my balance is very good and I think I need to do more to improve it… it sounds funny but I’ve found myself being nervous of walking on uneven surfaces, like cracked pavement slabs or even because I have fallen for no reason quite a few times. And I’m thinking why am I falling like this? Other people just seem to walk quite easily over this… Maybe it’s the high heels, whatever, I don’t know… even when I’m walking in like the right shoes or trainers, or walking shoes, I’m fine but I’m still very conscious, you know, of where I’m placing my feet, where I think when I was younger I wouldn’t care less, I’d just go for it”* Participant 4, Female, aged 56 years

*“I fell over once and I made sure ever since then, I’ve been so paranoid, that I take my time in whatever I’m doing in order not to have an accident or lose my balance… Because if you lose your balance and fall over you could break something and I don’t want any additional problems”* Participant 6, Female, aged 63 years

*“I have to be very particularly careful where I put my steps. Because sometimes you’ll be walking on a smooth bit and you’ll be talking and all of a sudden there’s a little, just a little slight difference and that can put me off-balance a little bit… I think that can happen to anyone but with me I am particularly very careful of that, or things like walking down the stairs. Twice I’ve missed a step down the stairs and I’ve hurt myself”* Participant 10, Female, aged 55 years

Some participants were aware that their balance had deteriorated over time, meaning that they had to make a conscious effort to adopt coping mechanisms:

*“I can’t turn round very quickly. Like if somebody comes behind me in the room and they speak to me and whereas one time I would just automatically just turn round, I have to think about it”* Participant 13, Male, aged 63 years

*“As I’ve got older I would say it’s things that you did without thinking about, you have to think about now”* Participant 24, Male, aged 60 years

Although most people related balance deterioration specifically to age-related decline, those participants with pre-existing medical conditions were also aware that other factors (for example, medication side-effects, visual disturbances, some Covid symptoms) might be affecting their balance. For these respondents, dealing with their balance issues became another part of managing their condition:

*“If I’m having a bad week I’ve got my daughter, I’ve got my sons, my mum as well at the weekends, so they’re there for extra support. But if I’m feeling okay I kind of just take my time around the house and do what I can”* Participant 17, Female, aged 55 years

*“All depending how I feel in the morning when I get up…When I don’t feel like I want to do it… I’m in pain”* Participant 13, Male, aged 63 years

### Managing and preventing balance issues

Participants who had experienced balance issues - or were knowledgeable about the benefits of balance-related activity to reduce the risk of developing problems - had adopted various means to help improve and/or maintain their balance. These included practising specific balance skills, doing exercise routines which include activities to develop balance and regular stretching and walking:

*“It’s just a flat piece of wood on the floor now but I’m going to try to get it raised a bit… and I just walk along it just to try and I don’t know if it’s going to help me but it’s to improve my balance”* Participant 1, Male, aged 55 years

*“I try to sort of, you know, balance on one leg… like balancing on one leg and sort of like stretch or, you know… you do one leg and you try and bend down… because it’s important I think to try and do these sort of things”* Participant 5, Male, aged 55 years

*“I’ve got some weights at home. They’re only them sort of like dumbbells, but you know, I’ll sit in the chair and I can do those. I’ll stand up and do those. You know, if I’m sitting down I’ll sort of like do, find little exercises to do sat down, where I can sort of like do my leg exercise. You can extend them and bring them back, extend them and bring them back… And that’s something you can do just sat down in the chair, you know, without even standing up. But if I’m stood up obviously you’ve got to have your balance and your stance to be able to operate your dumbbells without falling over. So that helps with your stamina, helps with your strength work, helps with your core as well, and it helps with your posture and being able to balance so you don’t fall over”* Participant 3, Female, aged 57 years

*“I know my balance isn’t that great, that’s why I do my stretching. And I’ve noticed as I’ve grown older, and I’m sure if I wasn’t doing my walking it would be really, really bad, but sometimes I find myself actually sort of tipping slow I don’t know. I can’t even explain it. Not walking straight. I know I’ve got a back problem but sometimes I feel as though I’m tipping to the side. It’s really weird but yeah. So anyway I’m sure that, you know, my stretching and then my walking helps towards hopefully my back, making sure my balance is okay”* Participant 8, Female, aged 57 years

One participant described making attempts to cover up their balance issues in public spaces whereas another reported using an aid to ensure that they felt safe when out and about:

*“I walk that strong and I try not to make nobody [notices] that I’m going to fall over because I’m losing my balance... I just sit down and just wait for it to come back”* Participant 16, Male, aged 55 years

*“If I’m in a supermarket I balance on the trolley. If I’m on the street, say going from a public car park to maybe a few shops on the high street, my local high street, I will take the stick with me because just in case I lose my balance. I have that extra security to balance on the stick. But indoors I don’t need a stick. Indoors I’m okay”* Participant 6, Female, aged 63 years

Several participants were not taking steps to improve their balance but reported being aware that they might benefit if they did. Some struggled to know which particular activities would develop their balance skills and one participant reported receiving conflicting advice from health professionals which had left her confused about which type of activities would work for her:

*“We do lots of family challenges like how long can you stand on one leg and different things. But yeah, I need to make it more of a focus, I feel that for myself”* Participant 4, Female, aged 56 years

*“Some exercises they give me is good for the lower back and some that they give me for the back is detrimental to the knee. So it’s like squats, yeah, and they’re telling me about squatting and doing it. I can’t handle the squatting. It then puts me out for about four days after that because I’ve grinded the knee. So you’re in a catch-22. I’ve got a situation where one particular exercise is good for one ailment and then it isn’t for another”* Participant 6, Female, aged 63 years

One participant put out a plea for any balance activities to be made interesting!

*“I’m quite an inpatient person and those types of exercises, just standing there, just going up and down, I just find a little bit boring. It’s not like I don’t know, I know that the net sum effect is going to be good, but it’s just I find, as I say, that a little bit boring”* Participant 11, Male, aged 55 years

## Discussion

We reported previously that among 50–70 year-olds there is a low level of knowledge and understanding of the ‘general’ physical activity recommendations (i.e. 150 min a week) [10]. This research suggests that not only is the knowledge of the strength and balance recommendation low (not a single respondent mentioned the recommendation to do strength and balance activities twice a week) but the perceived importance of strength and balance is also low. This is especially the case for balance activities.

Our study responded to the lack of primary qualitative research with English adults from black men and women. This was reported in two recent systematic reviews as a priority and a research gap. Ige-Elegbede et al’s 2019 review of ten UK qualitative studies reported only one study that included any African Caribbean participants [21]. This single study only had six participants participate in a focus group of eighteen in total [22]. Cavill et al’s 2021 systematic review again called for researchers to explore this research gap [13]. Cavill et al. 2021 also undertook a qualitative study (using the same methods as this study) and interviewed 58 adults from across England about their experiences and views of physical activity including strength and balance [12]. The study also reported that all participants rarely mentioned the importance of strength or balance training for older adults. Participants from a variety of ethnic backgrounds expressed a diverse and interesting range of views about physical activity, but the authors stated that it was notable how rarely they heard any reference to any cultural specific barriers or motivators. Our study has attempted to conduct in depth research with a specific minority ethnic group to tackle the paucity of primary research and under representation in this area.

As well as low levels of knowledge or understanding of the importance of strength and balance activities, there was low salience: most participants in our study did not think strength and balance activities played a very important part in their daily lives. They often conflated them with general exercise and bundled the benefits together under terms such as ‘use it or lose it’.

The public health recommendations around strength activities were devised based on evidence that there is an age-related decline in muscle strength, which can lead to a loss of function as older people lack the necessary strength to perform daily activities. A good example is the ability to be able to stand from sitting without using the arms: this is easy for most young people but is a marker of approaching disability in older people. This makes strength activities more important to promote as people age, not less. While participants in this study recognised the connection between strength and maintaining function, they did not generally seem to see this as something that needs more focus as we get older. Rather, participants were more likely to accept their declining strength and adapt their activities accordingly.

For balance the connection was made between good balance and independent living, but only when people had experienced a problem. So, participants seemed more likely to perform balance activities when they had had a fall (or a ‘wobble’) but not as a general preventative measure. A few participants mentioned the risk of falling as a stimulus for strength and balance and like Cavill et al’s 2021 paper, when it was referred to, it was by older participants, and in the context of more risky behaviours (slippery leaves; steep steps) rather than everyday walking or activities of daily living [12]. However, the salience of balance activities clearly increases with age as concerns about falling increase.

It is interesting to note that our participants were quite active for the age group (50–70) so it is possible that the salience of strength and balance activities may well be even lower among another sample of the same age group.

We deliberately interviewed a sample of people from the black community in England as this is a group that is under-represented in the literature, but we did not set out specifically to explore how ethnicity may affect views on strength and balance. However, we found very few references to culturally-determined issues on the topic, or to views or attitudes that may be unique to the black community.

The interviews provided limited clues from participants about how to increase knowledge of, and participation in, strength and balance activities. Most participants either viewed it as common sense, or common knowledge; or referred to specific advice they had been given – most frequently from an NHS professional (notably a physiotherapist) when they had experienced an injury. Some mentioned celebrity endorsement and fitness videos, not always in a positive sense, and this would probably reach a small minority of this age group. Collectively these findings suggest that there is a need for a multi-pronged programme to promote strength and balance activities among middle aged and older adults. This would need to include communication plans to raise awareness of the issue, perhaps supported by celebrity endorsers, discussion with NHS front line staff (including physiotherapists), as well as trusted information on how to make changes to daily lives. As previous evidence has shown that knowledge is a necessary first step but not sufficient to facilitate behaviour change there will also be a need to develop new behaviour change strategies to help older adults increase their strength and balance. Developing communication strategies and behaviour change approaches for this group are therefore key research priorities, as summarised in Table 2.

**Table 2:** Key findings and implications

Key findings	Implications
Only very general understanding of the importance of maintaining body strength and balance	Consider a communications programme specifically to emphasise the importance of strength and balance as one ages
Low salience: strength and balance activities were not seen to be an important part of participants’ lives	Perhaps emphasise the negative consequences (e.g. falls and loss of independence)
Most participants only want to be strong enough to get on with normal life	Specifically emphasise the importance of doing more as one ages (not less)
Participants aged 50–70 likely to think they were too young to worry about strength and balance	Encourage preparation for older age
Balance tended to be mentioned only if someone had experienced a problem	Emphasise the importance of good balance
NHS staff – especially physiotherapists – are a key source of information on the topic	Involve NHS staff in specific broad communications programme, along with the concept of ‘making every contact count’

## Strengths and limitations

The England wide sample was constructed to represent the voices of people who are often not heard in UK research and the focus on middle-aged black adults is a key strength of the study. We were unable to check back with interviewees about analysis of their interviews due to a lack of resource and capacity.

As it uses qualitative research methods, the findings of this study are not intended to be extrapolated to the general population as the sample did not set out to be representative. It is possible that this sample is more physically active than the average for this age group, which should be considered when interpreting the results. While we did engage with a population group that is often under-represented in UK research, we did not extend this to other ethnic minority communities in the UK. We would encourage researchers to repeat this study among the UK’s other main ethnic minority communities and identify through coproduction potential options for interventions development.

## Conclusion

Public health recommendations stress the importance of increasing participation in regular strength and balance activities as people age, to reduce the risk of falls and increase independence. This study has shown that among the black middle-aged adults we interviewed, the knowledge and salience of this message is low. Public health approaches should be taken to communicate the importance of enhancing strength and balance as people approach older age, including communication and education programmes, perhaps initially led, and delivered by health professionals, who have the highest authority with this topic. If we ignore this topic, it is possible that our ageing population in the UK will suffer from reduced independence and increased disability as they age.

## Data Availability

The datasets used and/or analysed during the current study are available from the corresponding author on reasonable request.

## Declarations

### Authors Information

Centre for Exercise, Nutrition and Health Sciences, School for Policy Studies, University of Bristol, 8 Priory Road, Bristol, BS8 1TZ, UK.

Nick Cavill, Gill Cowburn, Russell Jago & Charlie Foster.

National Institute for Health Research Bristol Biomedical Research Centre, University Hospitals Bristol and Weston NHS Foundation Trust and University of Bristol, Bristol, UK.

Russell Jago & Charlie Foster. Population Health Sciences, Bristol Medical School, University of Bristol, 5 Tyndall Avenue, BS8 1UD, UK.

Russell Jago.
